# Supplementary figures and images for: Spinocerebellar ataxia type 11-associated alleles of Ttbk2 dominantly interfere with ciliogenesis and cilium stability
Source: PLoS Genet. 2018 Dec 10;14(12):e1007844. doi: 10.1371/journal.pgen.1007844 (PMC6307817; doi:10.1371/journal.pgen.1007844)

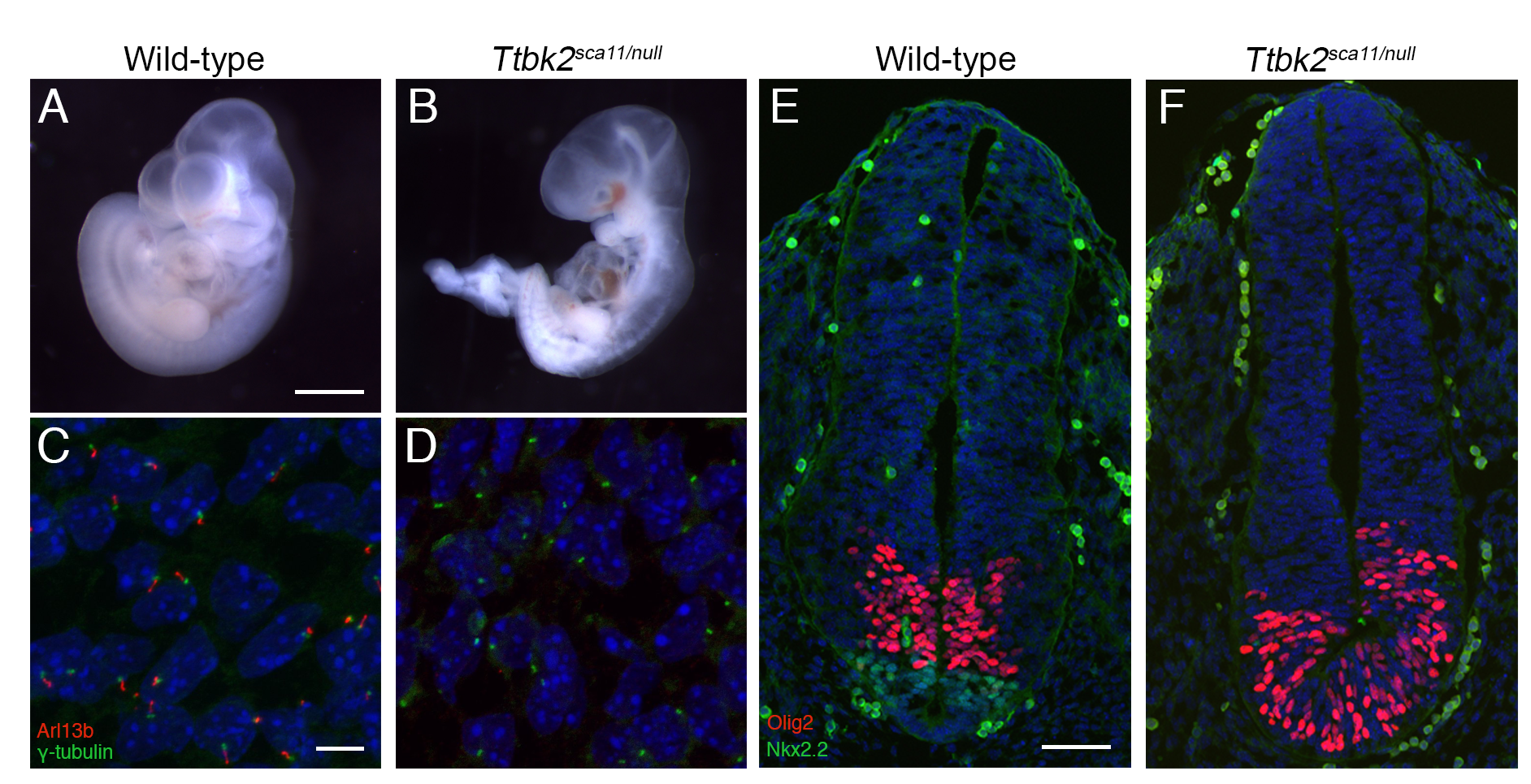

Supplement: S1 Fig — (A,B) Representative Wild type (A) and Ttbk2sca11/null (B) E10.5 embryos. Scale bar = 1mm. (C,D) Mesenchymal cells surrounding neural tube of E10.5 Wild-type (C) and Ttbk2sca11/null (D) embryos. Sections are immunostained for cilia using ARL13b (red) and γ-Tubulin (green). Scale bar = 20μm. (E,F) Transverse sections of E10.5 neural tubes of Wild-type (E) and Ttbk2sca11/null (F). Sections are immunostained for NKX2.2 to label V3 interneuron progenitors (green) and OLIG2 (red) to label motor neuron progenitors. Scale bar = 100μm. (TIF) [file pgen.1007844.s001.tif]

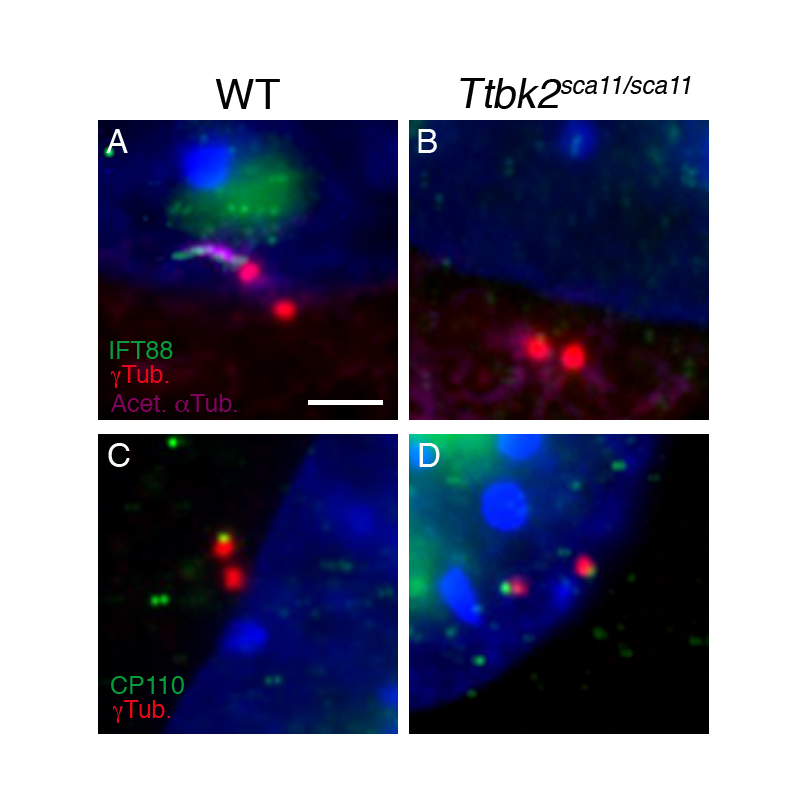

Supplement: S2 Fig — (A,B) MEFs of the indicated genotype were serum starved for 48 hours and immunostained for IFT88 (green) as well as γ-Tubulin (red) to label centrosomes and Acetylated α-Tubulin (magenta) to label the axonemes of cilia. Ttbk2sca11/sca11 cells lack cilia and also lack IFT88 at the mother centriole. (C,D). Serum starved MEFs were treated as above and stained for CP110 (green) and γ-Tubulin (red). Ttbk2sca11/sca11 cells retain CP110 on both centrosomes in the absence of serum. Scale bar = 5μm. (TIF) [file pgen.1007844.s002.tif]

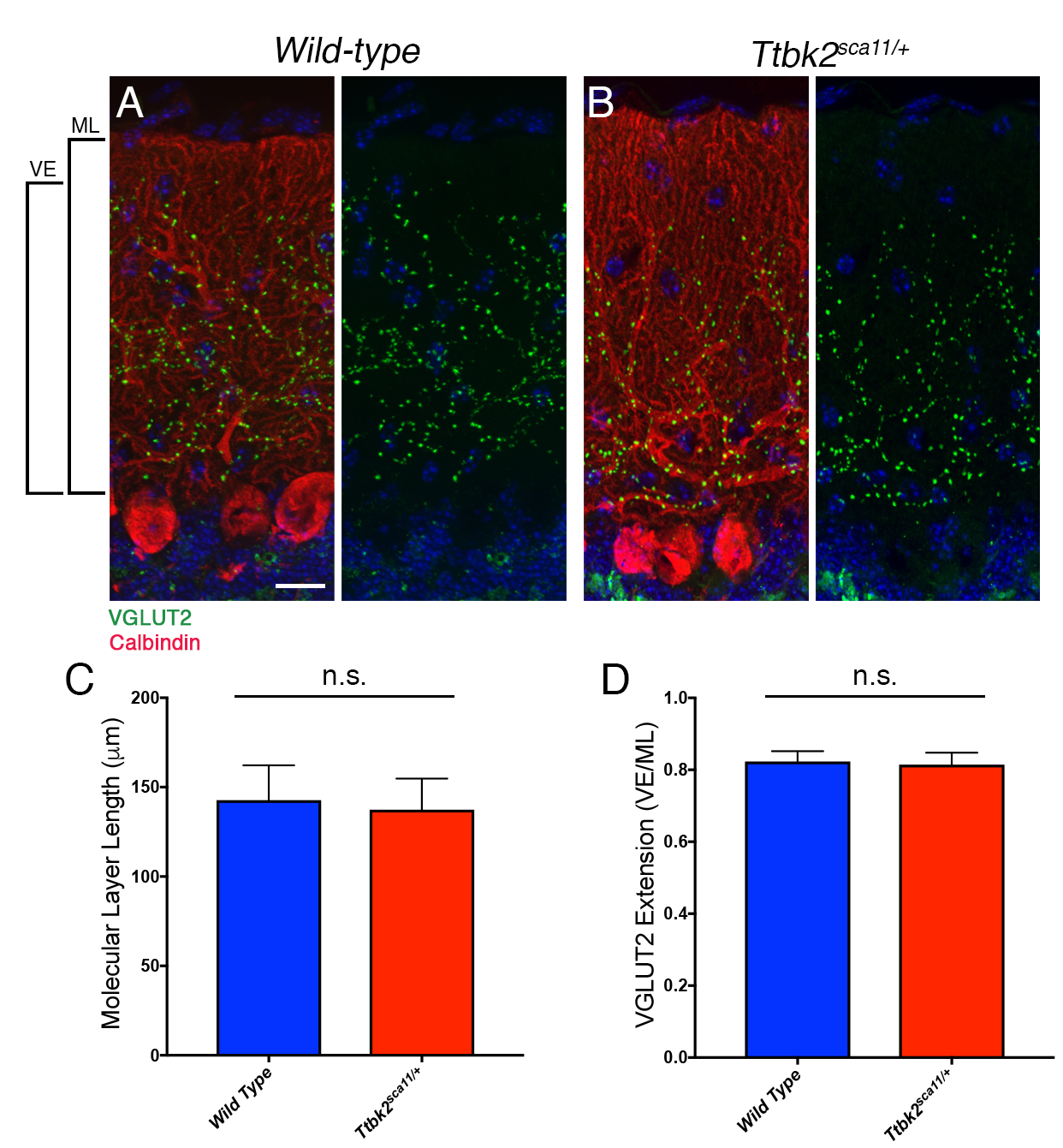

Supplement: S3 Fig — (A,B) Representative sagittal sections through the cerebellum of mice of the indicated genotype. Purkinje cells are labeled with Calbindin (red) and excitatory synapses from the climbing fibers onto the Purkinje cell dendrites are labeled with VGLUT2 (green). Scale bar = 50μm. (C,D) Measurements for the molecular layer thickness and VGLUT2 extension were made from four separate primary folia, pooled from three individual mice for each condition. Error bars denote SEM. The molecular layer thickness and VGLUT2 extension are unchanged between wild-type and Ttbk2sca11/+. (TIF) [file pgen.1007844.s003.tif]

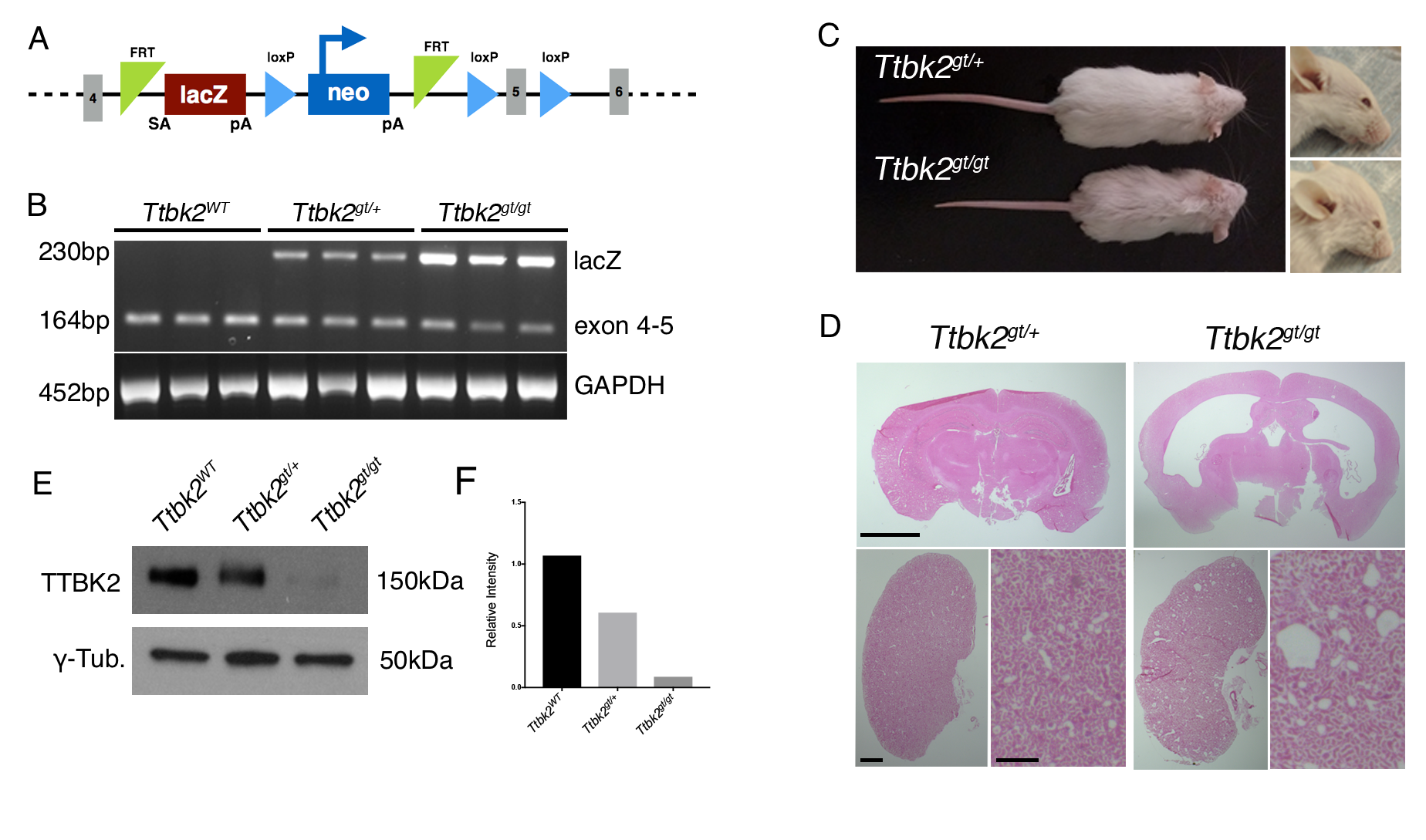

Supplement: S4 Fig — (A) Schematic of the Ttbk2 gene trap (Ttbk2gt) targeting design (schematic adapted from International Mouse Phenotyping Consortium). (B) RT-PCR analysis of WT splicing in Ttbk2gt/gt. RNA from 3 biological replicate brains per genotype was used, and primers targeting the exon 4–5 boundary show that WT transcript is still produced in Ttbk2gt/gt mice. (C) P30 mice showing phenotypic differences between Ttbk2gt/+ and Ttbk2gt/gt. (D) H&E staining of neural cortex and kidney tissue from 6mo old Ttbk2gt/gt mice showing hydrocephaly and polycyctic kidneys. Scale bar = 1mm. (E) Western blot showing decreased TTBK2 protein levels (150kDa) in lysates from Ttbk2WT, Ttbk2gt/+, and Ttbk2gt/gt brains. γ-Tubulin is a loading control. (F) Quantification of the relative intensity of the Western blot bands for TTBK2. Normalized TTBK2 protein levels in Ttbk2gt/gt brain lysate are 8.2% of the amount in Ttbk2WT brain lysate. (TIF) [file pgen.1007844.s004.tif]

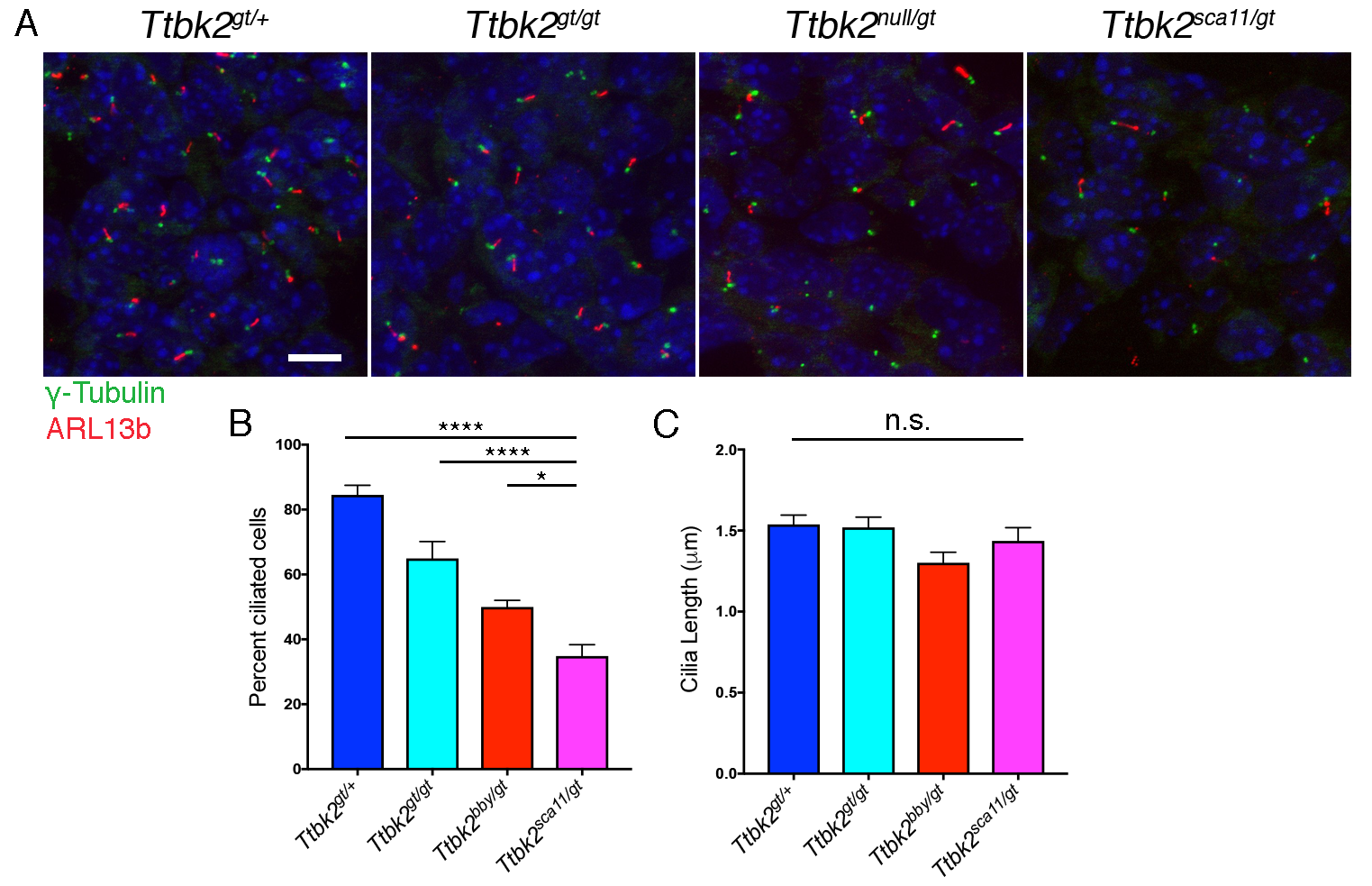

Supplement: S5 Fig — (A) Representative images of mesenchymal cells surrounding the neural tube of E10.5 embryos of the indicated genotype. Cilia were immunostained for ARL13b (red) to label cilia and γ-Tubulin (green) to label centrosomes. Scale bar = 20μm. (B) Quantification of the percentage of ciliated cells in the mesenchyme of the indicated genotype. Cilia are less abundant in Ttbk2sca11/gt embryos. Statistical comparison was performed by 1-way ANOVA with Tukey-Kramer post-hoc test. (p<0.0001 vs Ttbk2gt/+; p<0.0001 vs Ttbk2gt/gt; p = 0.0279 vs Ttbk2null/gt). n = two fields of view, three biological replicates, over 1000 total cells counted per genotype. (C) Quantification of cilia length in the mesenchyme of the indicated genotype. Cilia length is not statistically significantly different across the Ttbk2 allelic series in the embryonic mesenchyme. n = 50 cilia pooled from 3 biological replicates. (TIF) [file pgen.1007844.s005.tif]
