# Supplementary material for: Spinocerebellar ataxia type 11-associated alleles of Ttbk2 dominantly interfere with ciliogenesis and cilium stability
Source: PLoS Genet. 2018 Dec 10;14(12):e1007844. doi: 10.1371/journal.pgen.1007844 (PMC6307817; doi:10.1371/journal.pgen.1007844)
Supplement: S1 Table — (DOCX) [file pgen.1007844.s006.docx]

| Cross | Stage | Total Number | Number of mutants | Percent Mutant |
| --- | --- | --- | --- | --- |
| Null X Null | E9.5-E10.5 | 330 | 63 | 19.1 |
| Sca11 X Sca11 | E9.5-E10.5 | 94 | 20 | 21.3 |
| GT X GT | E12.5-E14.5 | 81 | 17 | 20.9 |
|  | E17.5-P0 | 183 | 44 | 24.0 |
| GT X Null | E12.5-E14.5 | 67 | 21 | 31.3 |
|  | E17.5-P0 | 43 | 8 | 18.6 |
| GT X SCA11 | E12.5-E14.5 | 99 | 22 | 22.2 |
|  | E17.5-P0 | 63 | 6 | 9.5 |

**Table S1. Numbers of mutants obtained from allelic series crosses.**
