# Supplementary material for: Spinocerebellar ataxia type 11-associated alleles of Ttbk2 dominantly interfere with ciliogenesis and cilium stability
Source: PLoS Genet. 2018 Dec 10;14(12):e1007844. doi: 10.1371/journal.pgen.1007844 (PMC6307817; doi:10.1371/journal.pgen.1007844)
Supplement: S2 Table — (DOCX) [file pgen.1007844.s007.docx]

| Cross | Stage | Number of mutants | Number polydactyly | Number holopros. | Number midbrain defect | Number forebrain defect |
| --- | --- | --- | --- | --- | --- | --- |
| Null X Null | E9.5-E10.5 | 63 | N/A | 63 | 63 | N/A |
| Sca11 X Sca11 | E9.5-E10.5 | 20 | N/A | 20 | 20 | N/A |
| GT X GT | E12.5+ | 61 | 4 | N/A | N/A | 0 |
| GT X Null | E9.5-E10.5 | 10 | N/A | 0 | 0 | N/A |
|  | E12.5+ | 29 | 29 | N/A | N/A | 19 |
| GT X SCA11 | E9.5-E10.5 | 12 | N/A | 4 | 4 | N/A |
|  | E12.5+ | 28 | 28 | N/A | N/A | 22 |

**Table S2. Summary of gross phenotypes observed from allelic series crosses.**
